# Biomarkers predictive of a response to extended endocrine therapy in breast cancer: a systematic review and meta-analysis

**DOI:** 10.1007/s10549-023-07149-x

**Published:** 2023-10-25

**Authors:** Kirsten M. Woolpert, Thomas P. Ahern, Timothy L. Lash, Donna L. O’Malley, Alice M. Stokes, Deirdre P. Cronin-Fenton

**Affiliations:** 1 Department of Clinical Epidemiology, Department of Clinical Medicine, Aarhus University and Aarhus University Hospital, Aarhus, Denmark; 2 Department of Surgery, The Robert Larner, M.D. College of Medicine at the University of Vermont, Burlington, VT USA; 3 Department of Epidemiology, Rollins School of Public Health, Emory University, Atlanta, GA USA; 4 University Libraries, University of Vermont, Burlington, VT USA

**Keywords:** Breast cancer, Predictive biomarkers, Extended endocrine therapy, Systematic review, Breast cancer index

## Abstract

**Purpose:**

Extension of adjuvant endocrine therapy beyond five years confers only modest survival benefit in breast cancer patients and carries risk of toxicities. This systematic review investigates the role of biomarker tests in predicting the clinical response to an extension of endocrine therapy.

**Methods:**

We searched Ovid MEDLINE, Ovid Embase, Global Index Medicus, and the Cochrane Central Register of Controlled Trials using an iterative approach to identify full-text articles related to breast cancer, endocrine therapy, and biomarkers.

**Results:**

Of the 1,217 unique reports identified, five studies were deemed eligible. Four investigated the Breast Cancer Index (BCI) assay in three distinct study populations. These studies consistently showed that BCI score was predictive of response to extended endocrine therapy among 1,946 combined patients, who were predominately non-Hispanic white and postmenopausal.

**Conclusions:**

Evidence in the setting of predictive tests for extended endocrine therapy is sparse. Most relevant studies investigated the use of BCI, but these study populations were largely restricted to a single age, race, and ethnicity group. Future studies should evaluate a variety of biomarkers in diverse populations. Without sufficient evidence, physicians and patients face a difficult decision in balancing the benefits and risks of endocrine therapy extension.

**Supplementary Information:**

The online version contains supplementary material available at 10.1007/s10549-023-07149-x.

## Introduction

Biomarkers are critical tools for predicting prognosis and guiding treatment in breast cancer. Common breast cancer biomarkers include hormone receptors (*e.g.,* estrogen receptors), which contribute to tumor subtyping [1, 2]. Women with tumors overexpressing the estrogen receptor (i.e., ER +) are recommended to take at least five years of adjuvant endocrine therapy (ET) [3]. Tamoxifen is guideline treatment for premenopausal women and an alternative to aromatase inhibitors for postmenopausal women. Aromatase inhibitors (i.e., anastrozole, letrozole, exemestane) are indicated only in postmenopausal women [3]. In a meta-analysis of clinical trials of five years of adjuvant ET in early-stage breast cancer, tamoxifen approximately halved recurrence rates [4], as did aromatase inhibitors in postmenopausal women [5]. Numerous clinical trials have confirmed these findings, providing a basis for the current guideline recommendation of at least five years of treatment [6].

Though the benefits of ET are pronounced, 20–40% of treated patients recur 5–20 years after diagnosis [7]. Recurrences have been documented even 39 years after primary diagnosis [8–10]. This hazard of late recurrence suggests a benefit of extending ET beyond the traditional five-year course. Several trials have shown a modest survival benefit and reduction of recurrence risk with extended ET depending on the duration, type, and sequence of the drugs [11, 12]. Other trials have found no improvement in overall survival with extended treatment [13–15]. The inconsistency in findings is further complicated as ET has long-term side effects. In the Adjuvant Tamoxifen, Longer Against Shorter (ATLAS) and the Adjuvant Tamoxifen-To Offer More (aTTom) trials—which evaluated extended tamoxifen use—there were increased risks of endometrial cancer and pulmonary embolism among women assigned to extended tamoxifen compared with placebo [11, 12]. Toxicities are also seen with long-term use of aromatase inhibitors, including increased risk of hypercholesterolemia, osteoporosis, fracture, and musculoskeletal syndrome [13–18].

Although clinical trials show that continuing ET beyond five years reduces late recurrence risk, it is essential to balance benefits with the risks of overtreatment using predictive and prognostic markers [19]. A prognostic biomarker informs the likelihood of a clinical outcome independent of any treatment received. In contrast, a predictive biomarker provides information on individuals most likely to respond to a specific treatment, differentiating patients likely to benefit from patients unlikely to benefit. To determine whether a biomarker is predictive, the study must include individuals who were treated (i.e., with extended ET), to compare them with untreated patients (i.e., those who stopped treatment after five years) [20]. There are several predictive and prognostic tests recommended by the American Society of Clinical Oncology (ASCO) and the US National Comprehensive Cancer Network (NCCN), such as OncotypeDx [21]. These tests characterize women by their risk of recurrence and have been pivotal in identifying low-risk patients who can forego chemotherapy [22–25].

The Breast Cancer Index (BCI) assay was developed in 2011 and is the only NCCN- and ASCO-approved test to predict benefit from extended ET [26]. The assay involves two parts: (1) the molecular grade index (MGI), a 5-gene predictor that measures tumor grade and proliferation and (2) the predictive panel, based on the expression ratio of HOXB13 and IL17BR (i.e., the H/I ratio or H/I) [27]. The BCI predictive panel stratifies patients into two groups: BCI (H/I) High, which indicates potential benefit from extended ET, and BCI (H/I) Low, which indicates low likelihood of benefit [21, 26–28]. The 2022 ASCO guideline update recommended that the BCI test be used to guide decisions about extended ET among ER + patients with node-negative disease or 1–3 positive nodes [26]. However, the evidence in premenopausal and perimenopausal women, and in those with > 3 positive lymph nodes, is limited. Predictors of early and late recurrences may differ according to menopausal status, generating a potential evidence gap in this setting [31]. Such information–perhaps provided by tumor biomarkers–could help patients and providers decide whether extending ET is worthwhile.

In this systematic review, we aimed to evaluate studies investigating biomarkers predictive of response to extended ET. Rather than focusing on late recurrence risk prediction, this review only involved populations treated with extended ET or standard duration treatment and in whom a predictive biomarker was assayed to predict response to the extended treatment.

## Methods

### Search strategy

We performed this review in accordance with the Preferred Reporting Items for Systematic Reviews and Meta-Analyses (PRISMA) guidelines [32]. Two medical librarians (DLO & AMS) performed a comprehensive search in consultation with the lead authors and informed by a Medical Subject Heading (MeSH) analysis. We used an iterative process to translate and refine searches in each database. We limited results to full-text peer-reviewed journal articles published in English. The formal search strategies used relevant terms and synonymous free text words and phrases to capture the concepts of breast cancer, extended ET, and biomarkers. Databases included MEDLINE (OvidSP), Embase (OvidSP), Global Index Medicus (WHO), and Cochrane Central Register of Controlled Trials (Wiley). The search covered January 1, 2006 through October 24, 2022. Detailed search strategies are outlined in the supplementary material.

One author (KMW) screened titles and abstracts of all papers. Full-text review and data extraction were conducted (KMW, DCF, TLL, & TPA) for consideration of inclusion. Studies were eligible if they included individuals treated with extended ET (i.e., treatment beyond five years after diagnosis) compared to a standard treatment course (i.e., five years) and assessed the utility of biomarkers in these settings. We defined a biomarker as any measurable characteristic “evaluated as an indicator of normal biological processes, pathogenic processes, or pharmacologic responses to a therapeutic intervention” [33, 34]. Clinicopathological measures (*e.g.,* tumor size) were not considered biomarkers. We also screened all references from eligible papers.

### Data extraction

Four authors (KMW, DCF, TLL, & TPA) extracted data from eligible studies. We recorded information on author, publication date, country, study design, study aim, population characteristics, inclusion and exclusion criteria, number of participants, number of recurrences, biomarker type, endpoint or outcome measure, variables controlled for, median follow-up time, number of participants within each recurrence risk category (i.e., BCI (H/I) High vs. BCI (H/I) Low), and the risk of developing recurrence with extended ET versus standard ET. Authors also performed quality assessment and recorded potential biases.

### Data synthesis

Figures and summary statistics were created using ‘ggplot’ [35] and ‘metafor’ [36] in R v4.0 (Vienna, Austria). Among studies investigating the same biomarker type, we examined statistical heterogeneity in findings using the I^2^ statistic. We pooled hazard ratios (HRs) and/or odds ratios (ORs) and their 95% confidence intervals (95% CI) using both fixed- and random-effect models.

## Results

### Study characteristics

**Fig. 1:**
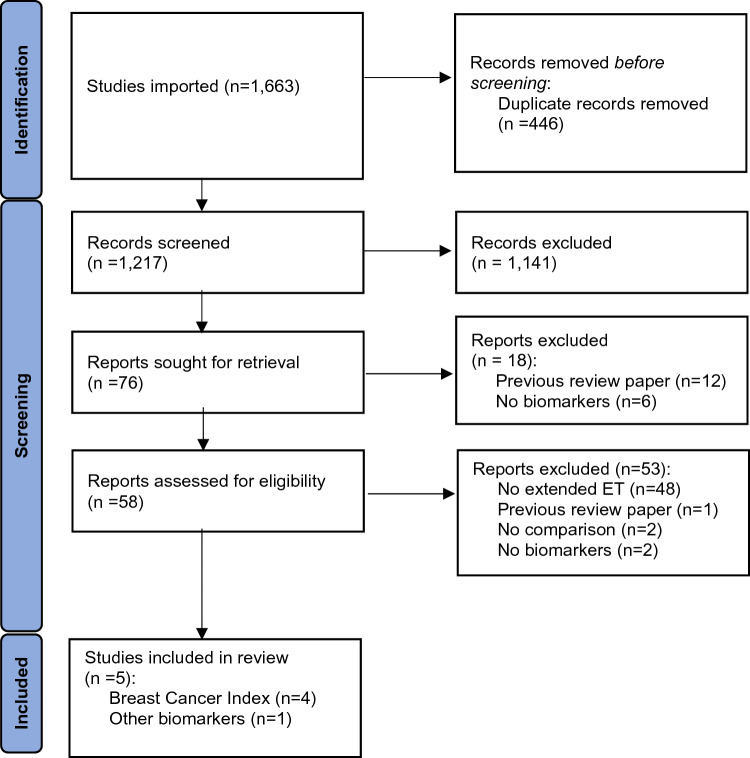
PRISMA 2020 flow diagram for the search, screening, and selection process of studies assessing biomarkers that may be predictive of a response to extended endocrine therapy among breast cancer patients

Our search yielded 1,663 articles, which were pooled to 1,217 unique reports. After title and abstract screening, 58 full-text articles had data extracted. Five studies were deemed eligible for inclusion: four investigated the utility of BCI and one investigated the utility of Ki67 and progesterone receptor (PgR) (Fig. 1). Eligible studies were published between 2013 and 2022 (Table 1). Inclusion criteria and participant information from eligible trials and analyses are outlined in Table 2.

**Table 1:** Characteristics of included studies investigating biomarkers for the prediction of extended endocrine therapy benefit

Study	Setting and diagnosis period	Study design	Parent trial population	Biomarker(s)	Comparison	Total patients analyzed	Number of patients with extended endocrine therapy (%)	Total number in each risk category (%)	Type of association estimate
Sgroi et al., 2013	North America, 1998–2003	Nested case–control	MA.17	BCI H/I	10 years of letrozole versus 5 years of letrozole + 5 years of placebo	249	122 (49)	H/I Low: 128 (51) H/I High: 121 (49)	Odds ratio estimate of rate ratio
Noordhoek et al., 2021	Netherlands, 2007–2011	Selected cohort from a randomized controlled trial	IDEAL	BCI H/I	10 years versus 7.5 years of letrozole	908	454 (50)	H/I Low: 479 (53) H/I High: 429 (47)	Hazard ratio
Bartlett et al., 2022	United Kingdom, 1991–2005	Selected cohort from a randomized controlled trial	Trans-aTTom	BCI H/I	10 years versus 5 years of tamoxifen	789	392 (50)	H/I Low: 385 (49) H/I High: 404 (51)	Hazard ratio
Villasco et al., 2021	Italy, 1988–2014	Cohort	–	Ki67; PgR	More than 5 years versus 5 years of endocrine therapy	783	180 (23)	Ki67 Low: 479 (61) Ki67 High: 304 (39) PgR + : 708 (90) PgR-: 75 (10)	Hazard ratio

**Table 2:** Major inclusion criteria and patient characteristics of studies eligible for this systematic review and meta-analysis

Parent trial characteristics						Extended endocrine therapy analysis			
Study	Parent trial population	Major inclusion criteria for parent trial	Total number of patients	Tumor stage at diagnosis [53]	Nodal status at diagnosis	Inclusion criteria for extended endocrine therapy analysis	Number of patients (% of trial participants)	Tumor stage at diagnosis	Nodal status at diagnosis
Sgroi et al., 2013	MA.17	Postmenopausal women with early-stage breast cancer with 5 years of tamoxifen completed	1918	T0: 1 (0.1) T1: 1,087 (57) T2: 647 (34) T3: 133 (6.9) T4, Tx: 50 (2.6)	N0: 894 (47) N1-N3: 986 (51) Nx: 38 (2.0)	All patients in parent trial with a recurrence and tumor tissue block, matched to two recurrence-free controls	249 (13%)	T1: 110 (44) T2: 111 (45) T3: 21 (8.4) T4,Tx: 7 (2.8)	N0: 94 (38) N1-N3: 146 (59) Nx: 9 (3.6)
Noordhoek et al., 2021	IDEAL	Postmenopausal women with early-stage breast cancer with five years of any adjuvant endocrine therapy completed	1718^a^	T1: 802 (47) T2: 774 (45) T3: 89 (4.9) T4, Tx: 53 (2.9)	N0: 461 (27) N1-N3: 1,248 (68) Nx: 9 (0.5)	All patients in parent trial with available tumor specimens	908 (50%)	T1: 406 (45) T2: 433 (48) T3: 49 (5.4) T4, Tx: 20 (2.2)	N0: 241 (27) N1-N3: 664 (73) Nx: 3 (0.3)
Bartlett et al., 2022	Trans-aTTom	Women with early-stage breast cancer with 5 years of tamoxifen completed	2445	T1: 1,510 (46) T2: 711 (43) T3: 52 (2.1) Tx: 172 (7.1)	N0: 1,367 (56) N1-N3: 789 (32) Nx: 289 (12)	All node-positive patients with available tumor specimens	789 (32%)	T1: 362 (46) T2: 336 (43) T3: 30 (3.8) Tx: 61 (7.8)	N0: 0 N1-N3: 789 (100) Nx: 0
Villasco et al., 2021	–	–	–	–	–	Patients treated for invasive breast cancer with complete clinical and pathological data	783	T1: 456 (58) T2: 301 (38) T3-T4: 26 (3.3)	N0: 462 (59) N1-N3: 321 (41)

^a^There were a total of 1824 patients in the IDEAL trial, but data provided by Noordhoek et al. used the parent cohort of 1,718 patients who were recurrence free at 2.5 years after randomization in the parent trial. Proportions in the extended endocrine therapy analysis reflect the total 1824 patients in the denominator

### Breast cancer index (4 articles)

#### MA.17 trial (1)

The MA.17 trial enrolled 1,918 postmenopausal, ER + breast cancer patients who were disease free after completing five years of adjuvant tamoxifen. Women were randomized to receive five years of extended letrozole or placebo [13]. Sgroi et al. conducted a nested case–control study and evaluated BCI in relation to late recurrence in patients from the MA.17 trial [23]. In this analysis, all patients with a recurrence and formalin-fixed, paraffin-embedded (FFPE) tumor tissue blocks were included and matched with two relapse-free controls, with a total of 249 patients analyzed. They found that individuals assigned to extended letrozole therapy and with a BCI (H/I) High score had improved recurrence-free interval (OR = 0.33, 95% CI 0.15, 0.73), which was less pronounced among those with a BCI (H/I) Low score (OR = 0.58, 95% CI 0.25, 1.36) when compared with individuals assigned to placebo [23].

These results are susceptible to selection bias, as only 100 of the 319 MA.17 recurrences had FFPE blocks available and authors further excluded patients with unknown or contralateral recurrence (*n* = 17 cases; 34 controls). Patients included in the nested case–control study were less likely to have radiation therapy and/or adjuvant chemotherapy and more likely to be older and have positive lymph nodes compared with the overall trial population. No information on cancer stage was available. Thus, the possibility of more advanced cancers in the study has the potential to influence findings.

#### Investigation on the duration of extended letrozole (IDEAL) trial (1)

The IDEAL study was a phase III-randomized, controlled trial of 1,824 postmenopausal, hormone receptor-positive patients. Women were randomized to receive either 2.5 or 5 years of letrozole after completing five years of ET [37]. Noordhoek et al. used all early-stage patients with available tumor specimens from this study (n = 908) to test BCI as a predictive marker of extended ET benefit. They found that, among BCI (H/I) High scoring patients, there was an improved recurrence-free interval among those with 5 years of extended letrozole compared to 2.5 years in the overall cohort (HR = 0.42, 95% CI: 0.21, 0.84). This was not seen among the BCI (H/I) Low scoring patients (HR = 0.95, 95% CI: 0.58, 1.56) [28]. The authors concluded that their findings “demonstrate significant prediction of extended endocrine benefit based on BCI (H/I) classification” [28].

This study is susceptible to selection bias because only ~ 50% of the original trial population was included. However, the authors did provide a table demonstrating well-balanced clinicopathological characteristics between the parent trial and the analyzed population. It is also important to note that the parent population was generally high risk. Thus, these results may not be generalizable to other populations.

#### The translational adjuvant tamoxifen-to offer more? (Trans-aTTom) trial (2)

The aTTom trial randomized 6,953 early breast cancer patients with ER + disease to receive either 5 or 10 years of tamoxifen [12]. These women were diagnosed from 1991 to 2005 from 176 medical centers across the UK and were followed up annually. The Trans-aTTom included patients in the original trial with available tumor blocks. Bartlett et al. evaluated the utility of BCI to predict benefit of 10 years of ET among a subset of node-positive patients in the Trans-aTTom trial population (*n* = 789). They found that, in the BCI (H/I) High stratum, patients randomized to 10 years of tamoxifen had an improved recurrence-free interval versus 5 years of tamoxifen (HR = 0.35, 95% CI 0.15, 0.86), with no benefit seen in the BCI (H/I) Low stratum (HR = 1.07, 95% CI 0.69, 1.65) [29]. In an article updating these results after completion of block collection, findings were similar (BCI (H/I) High HR = 0.33, 95% CI 0.14, 0.75; BCI (H/I) Low HR = 1.11, 95% CI 0.76, 1.64) [30].

Selection bias may threaten these results because tissue blocks were unavailable for approximately half of the original trial population. This analysis was also conducted only among the high-risk, node-positive women, as the study did not have sufficient power to investigate node-negative women. Additionally, this study was largely composed of postmenopausal women treated with long-term tamoxifen treatment, which is not reflective of the current guidelines for adjuvant ET.

### Other biomarkers (1 article)

Villasco et al. compared the Clinical Treatment Score-5 (CTS5) to other risk stratification methods [38]. Although CTS5 itself is not eligible for inclusion due to its exclusive reliance on clinicopathologic factors rather than on a biomarker, this study’s results regarding Ki67 level and PgR status are eligible. Villasco et al. selected patients treated between 1988 and 2014 for invasive breast cancer at one hospital in Italy who had complete clinicopathological and immunohistochemical data (*n* = 783 women).

#### Ki67

**Fig. 2:**
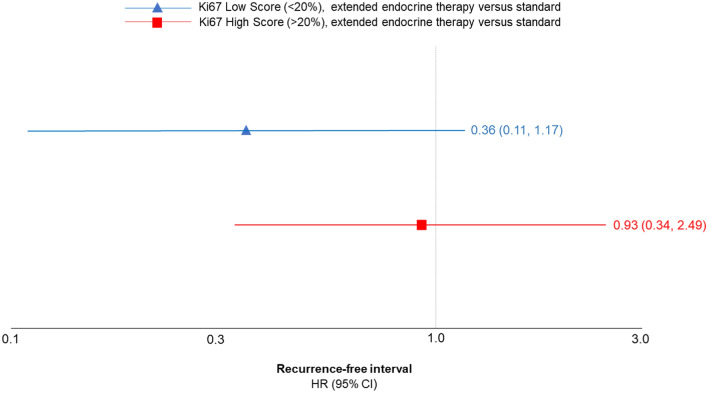
Association between extended endocrine therapy (> 5 years) compared with standard treatment and breast cancer recurrence-free interval, stratified by Ki67 score, Villasco et al. (2021) [38]

Ki67 is a marker of cellular proliferation frequently tested in breast cancer, where a high score represents a high rate of proliferation and thus more aggressive disease [39]. Villasco et al. dichotomized Ki-67 level at 20%, where individuals with < 20% positively stained tumor cells among the total number of assessed cells were considered low risk and > 20% were high risk. Among women with a low Ki67 level, the hazard of late distant recurrence was reduced among those treated with extended ET compared with just guideline ET (HR = 0.36, 95% CI 0.11, 1.17). In the same comparison among women with a high Ki67 level, the hazard of late distant recurrence did not differ between the two treatment groups (HR = 0.93, 95% CI 0.34, 2.49), indicating that there could be a predictive effect (Fig. 2) [38].

#### Progesterone receptor status

PgR is often tested in conjunction with ER and has been shown to improve clinical outcome prediction over testing ER status alone [40]. However, in the Villasco et al. study, PgR status did not predict extended ET benefit. Compared with five years of ET, women on extended treatment with PgR-positive tumors had a decreased hazard of late distant recurrence (HR = 0.56, 95% CI 0.26, 1.20), which did not meaningfully differ from the same comparison with PgR-negative tumors (HR = 0.78, 95% CI 0.09, 6.14]) [38].

This population again represented a selected sample, including only patients with complete data and follow-up. Additionally, of their total cohort of 783, only 180 extended ET (23%). Those with therapy extension were more likely to have larger and higher stage tumors compared with women who stopped treatment at five years. By not having a randomized sample and by not accounting for treatment differences, this study is susceptible to confounding, particularly by disease severity.

### Summary results

**Fig. 3:**
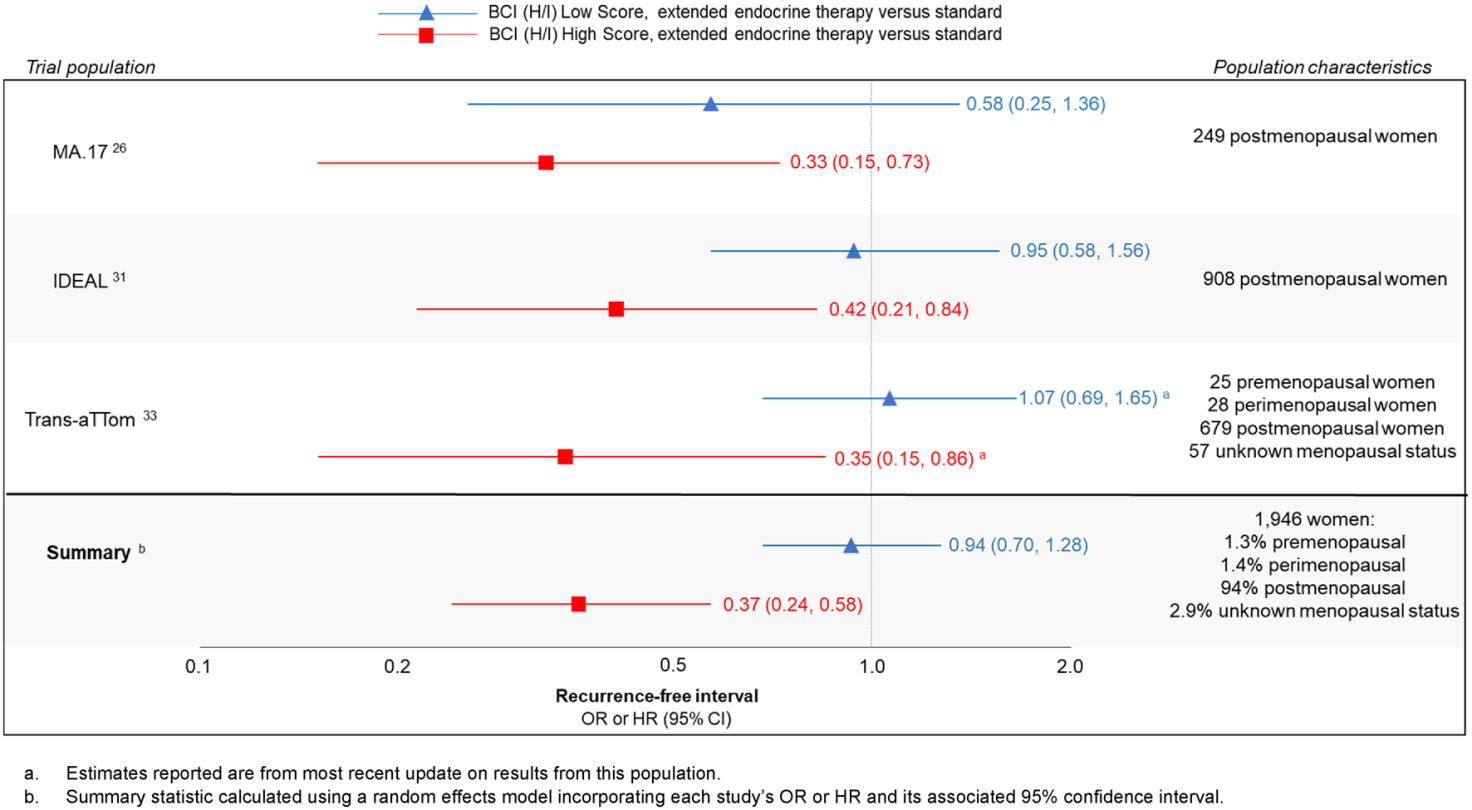
Associations between extended endocrine therapy (> 5 years) compared with standard treatment and breast cancer recurrence-free interval, stratified by Breast Cancer Index HOXB13/IL17BR (BCI (H/I) Score) in three distinct study populations. **a** Estimates reported are from most recent update on results from this population. **b** Summary statistic calculated using a random effects model incorporating each study’s OR or HR and its associated 95% confidence interval

Meta-analyses of results were only conducted for three BCI predictive studies—Sgroi et al., Noordhoek et al., and the latest publication from Bartlett et al. (Fig. 3) [23, 28, 30]. Little heterogeneity was seen in the three study results (*I*^2^ statistic < 1%). Thus, the fixed- and random-effects models yielded the same results. Patients with a BCI (H/I) Low score did not have a predicted benefit from extended ET compared with standard treatment with a pooled estimate of 0.94 (95% CI 0.70, 1.28). Conversely, those with a BCI (H/I) High score appeared to benefit from extended ET versus the standard treatment, with a pooled estimate of 0.37 (95% CI 0.24, 0.58).

## Discussion

In this review, four studies examined the utility of a biomarker in predicting clinical benefit from extended ET using the BCI Predictive assay and one study examined the predictive ability of Ki67 and PgR status. Of these, predictive capacity was seen for Ki67 in one study and for BCI Predictive assay in three distinct study populations. Though Villasco et al. concluded that no predictive response was seen with Ki67 risk stratification, there did appear to be a distinction in late distant recurrence risk comparing low versus high Ki67 level [38]. Remaining included studies consistently showed that a BCI (H/I) High score predicted benefit from extended ET, while a BCI (H/I) Low score did not. The BCI Predictive assay measures estrogenic and other proliferative signaling pathways in the progression of breast cancer, providing a risk-based score on the predicted benefit of additional therapy after completing the standard five years of ET [41]. However, the low number of eligible studies in this systematic review highlights the need for further research in this setting.

Our search identified many studies that investigated the utility of biomarkers in predicting overall late recurrence risk. Late recurrences occur when dormant cells remain inactive for some time, before reactivating to cause relapse. The underlying biology of dormancy remains poorly understood but is an active area of research. The Early Breast Cancer Trialists’ Collaborative Group (EBCTCG) periodically reviews the continued follow-up in trials such as ATLAS and aTTom to evaluate strategies for reducing late recurrence [7]. In EBCTCG’s latest study on late recurrence risk, clinicopathological features such as original tumor/lymph node status and Ki-67 status were predictive of recurrence from 5 to 20 years (level of evidence: 1B) [7, 42]. In this systematic review, late recurrence risk was often deemed a proxy for individuals who may benefit from treatment extension. These studies use evidence from biomarkers that stratify the risk for late recurrence, but this addresses a different question than that of predicting extended ET benefit. Though these studies may hint at vulnerable patients, they do not evaluate the predictive ability of the biomarker itself. Other molecular tests (*e.g.,* OncotypeDX) have shown some prognostic value in the setting of late recurrence, but are not recommended for decision-making due to the lack of predictive studies [26].

Recently published ASCO guidelines recommend BCI testing to assess potential benefit of extended ET in disease with negative nodes or 1–3 positive nodes. However, the recommendation is only supported by intermediate evidence quality and a moderate strength [26]. In the guideline, Andre et al. note that the collective evidence from five studies—three of which were identified in this systematic review (Bartlett et. al, 2019; Sgroi et. al, 2013; and Noordhoek et. al, 2021)—demonstrated a consistent predictive benefit of extended ET. Of the two studies not eligible for this review, one was published as an abstract only, so did not meet our a priori eligibility criteria [43]. Importantly, this study found that BCI (H/I) score was not predictive of an improvement in recurrence-free interval after extended letrozole therapy [43]. The second study that was ineligible for our review and cited in the ASCO guidelines investigated BCI in node-positive patients. Though the study included patients on extended therapy, it did not directly compare them to individuals who completed the guideline five years of therapy and thus did not evaluate predictive ability of the BCI (H/I) score [41]. The fourth study that was eligible in this review was published after release of the ASCO guidelines. In a changing landscape of treatments and biomarker testing availability, it is essential to generate more evidence to support these guidelines. For example, since the 2015 approval of the first cyclin-dependent kinase (CDK) 4/6 inhibitors, no study has investigated the combined role of these drugs in addition to extended ET [44, 45]. As treatments change and improve, we must continue to generate both trial-nested and real-world evidence to understand the dynamics of extended ET.

Our review also calls attention to the lack of generalizability resulting from features of the populations of included studies. One of the three studies—the Trans-aTTom trial—included premenopausal and perimenopausal women. However, these women only comprised about 8% of the population (*n* = 25 premenopausal; *n* = 28 perimenopausal) [32, 33]. ASCO guidelines state that their recommendations for the predictive ability of BCI ‘cannot definitively be made’ for premenopausal and perimenopausal women [26]. Additionally, cancer clinical trial participation has historically been predominately composed of non-Hispanic white and higher-income individuals, limiting the generalizability of findings [46]. For example, in the overall MA.17 trial, 91.9% of participants were non-Hispanic white women [13]. Not only are minority populations less likely to partake in clinical trials, but they are also less likely to receive biomarker testing [47]. In a meta-analysis of testing inequalities, lower socio-economic position was associated with a decrease in predictive biomarker test utilization (OR = 0.86, 95% CI 0.71, 1.05, 10 studies) [48]. The mean cost of the BCI Predictive assay is $3,450, and in the US is only covered by Medicare under certain criteria [49]. Given this high cost and lack of evidence in socio-economically disadvantaged populations, the recommendation of routine BCI testing is introducing what will inevitably be a disparity both in receiving these tests and in understanding their clinical utility in underrepresented populations.

Another theme in these studies was making inferences based on hypothesis tests of treatment/biomarker interaction terms. All BCI-related papers reported this statistic and used it as evidence supporting their conclusion of a predictive effect of BCI (H/I) score on extended ET response. In this test, a full model including an interaction term between BCI (H/I) score and extended ET is compared with a reduced model without an interaction term. A likelihood ratio test is used to determine whether the interaction term coefficient is statistically significantly different from zero based on a p-value threshold of 0.05. Significant interaction terms were interpreted as supporting the predictiveness of the BCI (H/I) test. In the context of log-linear models, this tests whether there is a departure from multiplicativity of effects, which is often difficult to interpret, particularly when evaluating the predictive ability of a biomarker [50]. If available, future studies should consider investigating departure from additive effects or using stratified estimates of effect to measure of interdependence [51]. Regardless, caution should be taken when interpreting results from likelihood ratio tests in this setting.

## Conclusion

This review outlines the limited research on biomarkers that predict a benefit from extended ET, including by use of commercially available tests, such as BCI. It is important to include premenopausal and perimenopausal women in future studies, as current studies in this area have nearly no representation of these important subpopulations of breast cancer patients who face the longest time at risk for recurrence. Additionally, diverse trial populations are essential, both because biomarker testing is differentially offered to many minority populations, but also because of lacking diverse trial. As breast cancer survival improves, the need to personalize treatment decisions will become increasingly important. Without sufficient evidence, healthcare teams and patients will face a difficult decision in balancing the benefits and risks of ET extension.

### Supplementary Information

Below is the link to the electronic supplementary material.

Supplementary file1 (DOCX 45 KB)

## Data Availability

All data generated or analyzed during this study are included in this article and its supplementary files.

## References

[1] Nofech-Mozes S, Vella ET, Dhesy-Thind S, Hagerty KL, Mangu PB, Temin S, Hanna WM (2012). Systematic Review on Hormone Receptor Testing in Breast Cancer. Appl Immunohistochem Mol Morphol.

[2] Polyak K (2011). Heterogeneity in breast cancer. J Clin Investig.

[3] Burstein HJ, Griggs JJ, Prestrud AA, Temin S (2010). American society of clinical oncology clinical practice guideline update on adjuvant endocrine therapy for women with hormone receptor-positive breast cancer. J Oncol Pract.

[4] Early Breast Cancer Trialists’ Collaborative Group (EBCTCG) (2011). Relevance of breast cancer hormone receptors and other factors to the efficacy of adjuvant tamoxifen: Patient-level meta-analysis of randomised trials. Lancet.

[5] Schneider R, Barakat A, Pippen J, Osborne C (2011). Aromatase inhibitors in the treatment of breast cancer in post-menopausal female patients: an update. Breast Cancer Targ Therapy.

[6] Burstein HJ, Lacchetti C, Anderson H, Buchholz TA, Davidson NE, Gelmon KA, Giordano SH, Hudis CA, Solky AJ, Stearns V, Winer EP, Griggs JJ (2019). Adjuvant endocrine therapy for women with hormone receptor-positive breast cancer: ASCO clinical practice guideline focused update. J Clin Oncol Off J Am Soc Clin Oncol.

[7] Pan H, Gray R, Braybrooke J, Davies C, Taylor C, McGale P, Peto R, Pritchard KI, Bergh J, Dowsett M, Hayes DF (2017). 20-year risks of breast-cancer recurrence after stopping endocrine therapy at 5 years. N Engl J Med.

[8] Pedersen RN, Esen BÖ, Mellemkjær L, Christiansen P, Ejlertsen B, Lash TL, Nørgaard M, Cronin-Fenton D (2022). The incidence of breast cancer recurrence 10–32 years after primary diagnosis. JNCI J Nat Cancer Insti.

[9] Mamby CC, Love RR, Heaney E (1993). Metastatic breast cancer 39 years after primary treatment. Wis Med J.

[10] Tashima Y, Kawano K (2014). A case of local recurrence developing thirty-nine years after mastectomy for breast cancer. Gan Kagaku Ryoho Cancer Chemother.

[11] Davies C, Pan H, Godwin J, Gray R, Arriagada R, Raina V, Abraham M, Alencar VHM, Badran A, Bonfill X, Bradbury J, Clarke M, Collins R, Davis SR, Delmestri A, Forbes JF, Haddad P, Hou M-F, Inbar M, Peto R (2013). Long-term effects of continuing adjuvant tamoxifen to 10 years versus stopping at 5 years after diagnosis of oestrogen receptor-positive breast cancer: ATLAS, a randomised trial. The Lancet.

[12] Gray RG, Rea D, Handley K, Bowden SJ, Perry P, Earl HM, Poole CJ, Bates T, Chetiyawardana S, Dewar JA, Fernando IN, Grieve R, Nicoll J, Rayter Z, Robinson A, Salman A, Yarnold J, Bathers S, Marshall A, Lee M (2013). aTTom: Long-term effects of continuing adjuvant tamoxifen to 10 years versus stopping at 5 years in 6,953 women with early breast cancer. J Clin Oncol.

[13] Goss PE, Ingle JN, Pritchard KI, Robert NJ, Muss H, Gralow J, Gelmon K, Whelan T, Strasser-Weippl K, Rubin S, Sturtz K, Wolff AC, Winer E, Hudis C, Stopeck A, Beck JT, Kaur JS, Whelan K, Tu D, Parulekar WR (2016). Extending aromatase-inhibitor adjuvant therapy to 10 years. N Engl J Med.

[14] Gnant M, Fitzal F, Rinnerthaler G, Steger GG, Greil-Ressler S, Balic M, Heck D, Jakesz R, Thaler J, Egle D, Manfreda D, Bjelic-Radisic V, Wieder U, Singer CF, Melbinger-Zeinitzer E, Haslbauer F, Sevelda P, Trapl H, Wette V, Greil R (2021). Duration of adjuvant aromatase-inhibitor therapy in postmenopausal breast cancer. N Engl J Med.

[15] Mamounas EP, Bandos H, Lembersky BC, Jeong J-H, Geyer CE, Rastogi P, Fehrenbacher L, Graham ML, Chia SK, Brufsky AM, Walshe JM, Soori GS, Dakhil SR, Seay TE, Wade JL, McCarron EC, Paik S, Swain SM, Wickerham DL, Wolmark N (2019). Use of letrozole after aromatase inhibitor-based therapy in postmenopausal breast cancer (NRG Oncology/NSABP B-42): A randomised, double-blind, placebo-controlled, phase 3 trial. Lancet Oncol.

[16] Monnier A (2006). Effects of adjuvant aromatase inhibitor therapy on lipid profiles. Expert Rev Anticancer Ther.

[17] Perez EA, Durling FC, Weilbaecher K (2006). Aromatase Inhibitors and Bone Loss. Oncol (Will Park NY).

[18] Hyder T, Marino CC, Ahmad S, Nasrazadani A, Brufsky AM (2021). Aromatase inhibitor-associated musculoskeletal syndrome: understanding mechanisms and management. Front Endocrinol.

[19] Hayes DF (2015). Clinical utility of genetic signatures in selecting adjuvant treatment: risk stratification for early vs. late recurrences. Breast.

[20] Ballman KV (2015). Biomarker: predictive or prognostic?. J Clin Oncol.

[21] Vieira AF, Schmitt F (2018). An update on breast cancer multigene prognostic tests—emergent clinical biomarkers. Front Med.

[22] Dubsky P, Filipits M, Jakesz R, Rudas M, Singer CF, Greil R, Dietze O, Luisser I, Klug E, Sedivy R, Bachner M, Mayr D, Schmidt M, Gehrmann MC, Petry C, Weber KE, Kronenwett R, Brase JC, Gnant M, Austrian Breast and Colorectal Cancer Study Group (ABCSG) (2013). EndoPredict improves the prognostic classification derived from common clinical guidelines in ER-positive, HER2-negative early breast cancer. Ann Oncol Off J Eur Soc Med Oncol.

[23] Sgroi DC, Carney E, Zarrella E, Steffel L, Binns SN, Finkelstein DM, Szymonifka J, Bhan AK, Shepherd LE, Zhang Y, Schnabel CA, Erlander MG, Ingle JN, Porter P, Muss HB, Pritchard KI, Tu D, Rimm DL, Goss PE (2013). Prediction of late disease recurrence and extended adjuvant letrozole benefit by the HOXB13/IL17BR biomarker. JNCI J Nat Cancer Instit.

[24] Filipits M, Nielsen TO, Rudas M, Greil R, Stöger H, Jakesz R, Bago-Horvath Z, Dietze O, Regitnig P, Gruber-Rossipal C, Müller-Holzner E, Singer CF, Mlineritsch B, Dubsky P, Bauernhofer T, Hubalek M, Knauer M, Trapl H, Fesl C, Austrian Breast and Colorectal Cancer Study Group (2014). The PAM50 risk-of-recurrence score predicts risk for late distant recurrence after endocrine therapy in postmenopausal women with endocrine-responsive early breast cancer. Clin Cancer Res Off J Am Assoc Cancer Res.

[25] Wolmark N, Mamounas EP, Baehner FL, Butler SM, Tang G, Jamshidian F, Sing AP, Shak S, Paik S (2016). Prognostic impact of the combination of recurrence score and quantitative estrogen receptor expression (ESR1) on predicting late distant recurrence risk in estrogen receptor-positive breast cancer after 5 years of tamoxifen: results from NRG oncology/national surgical adjuvant breast and bowel project B-28 and B-14. J Clin Oncol.

[26] Andre F, Ismaila N, Allison KH, Barlow WE, Collyar DE, Damodaran S, Henry NL, Jhaveri K, Kalinsky K, Kuderer NM, Litvak A, Mayer EL, Pusztai L, Raab R, Wolff AC, Stearns V (2022). Biomarkers for Adjuvant Endocrine and Chemotherapy in Early-Stage Breast Cancer: ASCO Guideline Update. J Clin Oncol.

[27] Jerevall P-L, Ma X-J, Li H, Salunga R, Kesty NC, Erlander MG, Sgroi DC, Holmlund B, Skoog L, Fornander T, Nordenskjöld B, Stål O (2011). Prognostic utility of HOXB13:IL17BR and molecular grade index in early-stage breast cancer patients from the Stockholm trial. Br J Cancer.

[28] Noordhoek I, Treuner K, Putter H, Zhang Y, Wong J, Meershoek-Klein Kranenbarg E, Duijm-de Carpentier M, van de Velde CJH, Schnabel CA, Liefers G-J (2021). Breast cancer index predicts extended endocrine benefit to individualize selection of patients with HR+ early-stage breast cancer for 10 years of endocrine therapy. Clin Cancer Res.

[29] Bartlett JMS, Sgroi DC, Treuner K, Zhang Y, Ahmed I, Piper T, Salunga R, Brachtel EF, Pirrie SJ, Schnabel CA, Rea DW (2019). Breast Cancer Index and prediction of benefit from extended endocrine therapy in breast cancer patients treated in the Adjuvant Tamoxifen—To Offer More? (ATTom) trial. Ann Oncol.

[30] Bartlett JMS, Sgroi DC, Treuner K, Zhang Y, Piper T, Salunga RC, Ahmed I, Doos L, Thornber S, Taylor KJ, Brachtel EF, Pirrie SJ, Schnabel CA, Rea DW (2022). Breast cancer index is a predictive biomarker of treatment benefit and outcome from extended tamoxifen therapy: final analysis of the trans-aTTom study. Clin Cancer Res Off J Am Assoc Cancer Res.

[31] Yamashita H, Ogiya A, Shien T, Horimoto Y, Masuda N, Inao T, Osako T, Takahashi M, Endo Y, Hosoda M, Ishida N, Horii R, Yamazaki K, Miyoshi Y, Yasojima H, Tomioka N, Collaborative Study Group of Scientific Research of the Japanese Breast Cancer Society (2016). Clinicopathological factors predicting early and late distant recurrence in estrogen receptor-positive, HER2-negative breast cancer. Breast Cancer.

[32] Page MJ, McKenzie JE, Bossuyt PM, Boutron I, Hoffmann TC, Mulrow CD, Shamseer L, Tetzlaff JM, Akl EA, Brennan SE, Chou R, Glanville J, Grimshaw JM, Hróbjartsson A, Lalu MM, Li T, Loder EW, Mayo-Wilson E, McDonald S, Moher D (2021). The PRISMA 2020 statement: an updated guideline for reporting systematic reviews. Syst Rev.

[33] Biomarkers Definitions Working Group (2001). Biomarkers and surrogate endpoints: preferred definitions and conceptual framework. Clini Pharm Ther.

[34] Strimbu K, Tavel JA (2010). What are Biomarkers?. Curr Opin HIV AIDS.

[35] Wickham H (2009). ggplot2: Elegant Graphics for Data Analysis. Springer.

[36] Viechtbauer W (2010). Conducting Meta-Analyses in R with the metafor Package. J Statist Soft.

[37] Blok, E. J., Kroep, J. R., Meershoek-Klein Kranenbarg, E., Duijm-de Carpentier, M., Putter, H., van den Bosch, J., Maartense, E., van Leeuwen-Stok, A. E., Liefers, G.-J., Nortier, J. W. R., Rutgers, E. J. T., van de Velde, C. J. H., (2018). Optimal Duration of Extended Adjuvant Endocrine Therapy for Early Breast Cancer; Results of the IDEAL Trial (BOOG 2006–05). JNCI J Nat Cancer Instit 110: 1 40–48 10.1093/jnci/djx134 28922787

[38] Villasco A, Accomasso F, D’Alonzo M, Agnelli F, Sismondi P, Biglia N (2021). Evaluation of the ability of the Clinical Treatment Score at 5 years (CTS5) compared to other risk stratification methods to predict the response to an extended endocrine therapy in breast cancer patients. Breast Cancer (Tokyo, Japan).

[39] Nielsen TO, Leung SCY, Rimm DL, Dodson A, Acs B, Badve S, Denkert C, Ellis MJ, Fineberg S, Flowers M, Kreipe HH, Laenkholm A-V, Pan H, Penault-Llorca FM, Polley M-Y, Salgado R, Smith IE, Sugie T, Bartlett JMS, Hayes DF (2020). Assessment of Ki67 in breast cancer: updated recommendations from the international Ki67 in breast cancer working group. JNCI J Nat Cancer Instit.

[40] Bardou V-J, Arpino G, Elledge RM, Osborne CK, Clark GM (2003). Progesterone receptor status significantly improves outcome prediction over estrogen receptor status alone for adjuvant endocrine therapy in two large breast cancer databases. J Clin Oncol.

[41] Zhang Y, Schroeder BE, Jerevall P-L, Ly A, Nolan H, Schnabel CA, Sgroi DC (2017). A novel breast cancer index for prediction of distant recurrence in HR+ early-stage breast cancer with one to three positive nodes. Clin Cancer Res.

[42] Burns PB, Rohrich RJ, Chung KC (2011). The levels of evidence and their role in evidence-based medicine. Plast Reconstr Surg.

[43] Mamounas EP, Bandos H, Rastogi P, Zhang Y, Treuner K, Lucas PC, Geyer CE, Fehrenbacher L, Graham M, Chia SKL, Brufsky A, Walshe JM, Soori GS, Dakhil SR, Paik S, Swain SM, Sgroi D, Schnabel CA, Wolmark N (2021). Breast Cancer Index (BCI) and prediction of benefit from extended aromatase inhibitor (AI) therapy (tx) in HR+ breast cancer: NRG oncology/NSABP B-42. J Cli Oncol.

[44] Hermansyah D, Firsty NN, Alhudawy MN, Nasution RA (2022). The Combination of CDK 4/6 Inhibitors plus Endocrine Treatment versus Endocrine Treatment Alone in Hormone-receptor (HR)-Positive breast Cancer: A Systematic Review and Meta-analysis. Medical Archives.

[45] Finn RS, Crown JP, Lang I, Boer K, Bondarenko IM, Kulyk SO, Ettl J, Patel R, Pinter T, Schmidt M, Shparyk Y, Thummala AR, Voytko NL, Fowst C, Huang X, Kim ST, Randolph S, Slamon DJ (2015). The cyclin-dependent kinase 4/6 inhibitor palbociclib in combination with letrozole versus letrozole alone as first-line treatment of oestrogen receptor-positive, HER2-negative, advanced breast cancer (PALOMA-1/TRIO-18): a randomised phase 2 study. The Lancet Oncol.

[46] Clark LT, Watkins L, Piña IL, Elmer M, Akinboboye O, Gorham M, Jamerson B, McCullough C, Pierre C, Polis AB, Puckrein G, Regnante JM (2019). Increasing diversity in clinical trials: overcoming critical barriers. Curr Probl Cardiol.

[47] Blok EJ, Bastiaannet E, van den Hout WB, Liefers GJ, Smit VTHBM, Kroep JR, van de Velde CJH (2018). Systematic review of the clinical and economic value of gene expression profiles for invasive early breast cancer available in Europe. Cancer Treat Rev.

[48] Norris RP, Dew R, Sharp L, Greystoke A, Rice S, Johnell K, Todd A (2020). Are there socio-economic inequalities in utilization of predictive biomarker tests and biological and precision therapies for cancer? A systematic review and meta-analysis. BMC Med.

[49] Ordering Breast Cancer Index Test. (n.d.). Retrieved February 1, 2023, from https://www.breastcancerindex.com/order-test

[50] Siemiatycki J, Thomas DC (1981). Biological models and statistical interactions: an example from multistage carcinogenesis. Int J Epidemiol.

[51] Rothman KJ, Lash TL, Greenland S, Rothman KJ, Greenland S, Lash TL (2008). Concepts of Interaction. Modern Epidemiology.

