# Supplementary material for: Biomarkers predictive of a response to extended endocrine therapy in breast cancer: a systematic review and meta-analysis
Source: Breast Cancer Res Treat. 2023 Oct 25;203(3):407–17. doi: 10.1007/s10549-023-07149-x (PMC10806232; doi:10.1007/s10549-023-07149-x)
Supplement: Supplementary file 1 — Supplementary file1 (DOCX 45 KB) [file 10549_2023_7149_MOESM1_ESM.docx]

**Supplementary Material**

**PRISMA 2020 checklist:**

| **Section and Topic** | **Item #** | **Checklist item** | **Location where item is reported** |
| --- | --- | --- | --- |
| **TITLE** | | |  |
| Title | 1 | Identify the report as a systematic review. | Title |
| **ABSTRACT** | | |  |
| Abstract | 2 | See the PRISMA 2020 for Abstracts checklist. |  |
| **INTRODUCTION** | | |  |
| Rationale | 3 | Describe the rationale for the review in the context of existing knowledge. | Introduction |
| Objectives | 4 | Provide an explicit statement of the objective(s) or question(s) the review addresses. | Introduction |
| **METHODS** | | |  |
| Eligibility criteria | 5 | Specify the inclusion and exclusion criteria for the review and how studies were grouped for the syntheses. | Methods – search strategy |
| Information sources | 6 | Specify all databases, registers, websites, organisations, reference lists and other sources searched or consulted to identify studies. Specify the date when each source was last searched or consulted. | Methods- search strategy |
| Search strategy | 7 | Present the full search strategies for all databases, registers and websites, including any filters and limits used. | Supplement |
| Selection process | 8 | Specify the methods used to decide whether a study met the inclusion criteria of the review, including how many reviewers screened each record and each report retrieved, whether they worked independently, and if applicable, details of automation tools used in the process. | Methods – search strategy |
| Data collection process | 9 | Specify the methods used to collect data from reports, including how many reviewers collected data from each report, whether they worked independently, any processes for obtaining or confirming data from study investigators, and if applicable, details of automation tools used in the process. | Methods – search strategy |
| Data items | 10a | List and define all outcomes for which data were sought. Specify whether all results that were compatible with each outcome domain in each study were sought (e.g. for all measures, time points, analyses), and if not, the methods used to decide which results to collect. | Methods – data extraction |
|  | 10b | List and define all other variables for which data were sought (e.g. participant and intervention characteristics, funding sources). Describe any assumptions made about any missing or unclear information. | Methods – data extraction |
| Study risk of bias assessment | 11 | Specify the methods used to assess risk of bias in the included studies, including details of the tool(s) used, how many reviewers assessed each study and whether they worked independently, and if applicable, details of automation tools used in the process. | Methods – data extraction |
| Effect measures | 12 | Specify for each outcome the effect measure(s) (e.g. risk ratio, mean difference) used in the synthesis or presentation of results. | Methods – data synthesis |
| Synthesis methods | 13a | Describe the processes used to decide which studies were eligible for each synthesis (e.g. tabulating the study intervention characteristics and comparing against the planned groups for each synthesis (item #5)). | Methods – data synthesis |
|  | 13b | Describe any methods required to prepare the data for presentation or synthesis, such as handling of missing summary statistics, or data conversions. | Methods – data synthesis |
|  | 13c | Describe any methods used to tabulate or visually display results of individual studies and syntheses. | Methods – data synthesis |
|  | 13d | Describe any methods used to synthesize results and provide a rationale for the choice(s). If meta-analysis was performed, describe the model(s), method(s) to identify the presence and extent of statistical heterogeneity, and software package(s) used. | Methods – data synthesis |
|  | 13e | Describe any methods used to explore possible causes of heterogeneity among study results (e.g. subgroup analysis, meta-regression). | NA |
|  | 13f | Describe any sensitivity analyses conducted to assess robustness of the synthesized results. | NA |
| Reporting bias assessment | 14 | Describe any methods used to assess risk of bias due to missing results in a synthesis (arising from reporting biases). | NA |
| Certainty assessment | 15 | Describe any methods used to assess certainty (or confidence) in the body of evidence for an outcome. | NA |
| **RESULTS** | | |  |
| Study selection | 16a | Describe the results of the search and selection process, from the number of records identified in the search to the number of studies included in the review, ideally using a flow diagram. | Results- study characteristics |
|  | 16b | Cite studies that might appear to meet the inclusion criteria, but which were excluded, and explain why they were excluded. | NA (similar articles in discussion) |
| Study characteristics | 17 | Cite each included study and present its characteristics. | Results |
| Risk of bias in studies | 18 | Present assessments of risk of bias for each included study. | Results |
| Results of individual studies | 19 | For all outcomes, present, for each study: (a) summary statistics for each group (where appropriate) and (b) an effect estimate and its precision (e.g. confidence/credible interval), ideally using structured tables or plots. | Results/Figures |
| Results of syntheses | 20a | For each synthesis, briefly summarise the characteristics and risk of bias among contributing studies. | Results |
|  | 20b | Present results of all statistical syntheses conducted. If meta-analysis was done, present for each the summary estimate and its precision (e.g. confidence/credible interval) and measures of statistical heterogeneity. If comparing groups, describe the direction of the effect. | Figures |
|  | 20c | Present results of all investigations of possible causes of heterogeneity among study results. | Figures |
|  | 20d | Present results of all sensitivity analyses conducted to assess the robustness of the synthesized results. | NA |
| Reporting biases | 21 | Present assessments of risk of bias due to missing results (arising from reporting biases) for each synthesis assessed. | Results |
| Certainty of evidence | 22 | Present assessments of certainty (or confidence) in the body of evidence for each outcome assessed. | Results |
| **DISCUSSION** | | |  |
| Discussion | 23a | Provide a general interpretation of the results in the context of other evidence. | Discussion |
|  | 23b | Discuss any limitations of the evidence included in the review. | Discussion |
|  | 23c | Discuss any limitations of the review processes used. | Discussion |
|  | 23d | Discuss implications of the results for practice, policy, and future research. | Discussion |
| **OTHER INFORMATION** | | |  |
| Registration and protocol | 24a | Provide registration information for the review, including register name and registration number, or state that the review was not registered. | Methods – search strategy |
|  | 24b | Indicate where the review protocol can be accessed, or state that a protocol was not prepared. | NA |
|  | 24c | Describe and explain any amendments to information provided at registration or in the protocol. | NA |
| Support | 25 | Describe sources of financial or non-financial support for the review, and the role of the funders or sponsors in the review. | Title page |
| Competing interests | 26 | Declare any competing interests of review authors. | Title page |
| Availability of data, code and other materials | 27 | Report which of the following are publicly available and where they can be found: template data collection forms; data extracted from included studies; data used for all analyses; analytic code; any other materials used in the review. | Supplement |

*From:*  Page MJ, McKenzie JE, Bossuyt PM, Boutron I, Hoffmann TC, Mulrow CD, et al. The PRISMA 2020 statement: an updated guideline for reporting systematic reviews. BMJ 2021;372:n71. doi: 10.1136/bmj.n71

For more information, visit: <http://www.prisma-statement.org/>

**Detailed search strategies:**

Database: Ovid MEDLINE(R) All

(exp Breast neoplasms/ or (breast neoplasm* or breast cancer).mp.) and (Antineoplastic Agents, Hormonal/ or exp Tamoxifen/ or (tamoxifen or ici-47699 or Nolvadex or Novaldex or Tomaxithen or zitazonium or ici-46474 or ici46474 or ici47699 or ici-46,474 or ici46,474 or soltamox).mp. or exp Aromatase inhibitors/ or (aromatase inhibitor* or 3,4-epoxy-5alpha-androstan-17-one or Aminoglutethimide or Anastrozole or Exemestane or Fadrozole or Formestane or Intraovarian peptides or Letrozole or Plomestane or Vorozole).mp. or exp Selective estrogen receptor modulators/ or ((exten* adj1 endocrine therapy) or selective estrogen receptor modulator* or SERM).mp.) and (exp Neoplasm recurrence, Local/ or Disease-free survival/ or Recurrence/ or Progression-free survival/ or (((endocrine therapy or tamoxifen or aromatase inhibitor*) adj1 resistan*) or disease free survival or breast cancer free interval or recurren*).mp.) and ((PAM50 or (PAM adj1 "50") or Prosigna or endopredict or epclin or clinical treatment score or 4-marker immunohistochemical score or IHC4* or MammaPrint or Mammostrat or OncoMas TR or Oncomastr or OMclin1 or Omclin2 or oncotype DX or CTSS or breast cancer index or HOXB13?IL17BR or (HOXB13 adj1 IL17BR) or ((treatment or recurrence or ROR or decision or propensity or prognostic or predict*) adj3 (score or panel or nomogram)) or ((biomarker* or marker*) adj2 (risk or survival or recurren* or failure or cancer-free or prevent* or prognos* or predict*))).mp.) and (eng.la.)

Limit to 2006-Current

**Database: Cochrane Central Register of Controlled Trials**

(Breast neoplasms or breast neoplasm* or breast cancer):ti,ab,kw AND (Antineoplastic Agents, Hormonal or tamoxifen or (ici NEXT 47699) or Nolvadex or Novaldex or Tomaxithen or zitazonium or (ici NEXT 46474) or ici46474 or ici47699 or (ici NEXT 46,474) or ici46,474 or soltamox or aromatase inhibitor* or (3,4 NEXT epoxy NEXT 5alpha NEXT androstan NEXT 17 NEXT one) or Aminoglutethimide or Anastrozole or Exemestane or Fadrozole or Formestane or Intraovarian peptides or Letrozole or Plomestane or Vorozole or Selective estrogen receptor modulators or (exten* NEAR/1 endocrine therapy) or selective estrogen receptor modulator* or SERM):ti,ab,kw AND (Neoplasm recurrence, Local or (Disease NEXT free survival) or (Progression NEXT free survival) or ((endocrine therapy or tamoxifen or aromatase inhibitor*) NEAR/1 resistan*) or breast cancer free interval or recurren*):ti,ab,kw AND (PAM50 or (PAM NEXT "50") or Prosigna or endopredict or epclin or clinical treatment score or (4 NEXT marker immunohistochemical score) or IHC4* or MammaPrint or Mammostrat or OncoMas TR or Oncomastr or OMclin1 or Omclin2 or oncotype DX or CTSS or breast cancer index or HOXB13?IL17BR or (HOXB13 NEXT IL17BR) or ((treatment or recurrence or ROR or decision or propensity or prognostic or predict*) NEXT/3 (score or panel or nomogram*)) or ((biomarker* or marker*) NEAR/2 (risk or survival or recurren* or failure or cancer NEXT free or prevent* or prognos* or predict*))):ti,ab,kw

**Database: Global Index Medicus**

| #1 | (mh:(breast neoplasms)) OR (tw:(breast neoplasm)) OR (tw:(breast neoplasms)) OR (tw:(breast cancer)) |
| --- | --- |
| #2 | (tw:((mh:("Antineoplastic Agents, Hormonal")) or (mh:(tamoxifen)) OR (tw:(tamoxifen)) OR (tw:(nolvadex)) OR (tw:(novaldex)) OR (tw:(tamoxifen citrate)) OR (tw:(tomaxithen)) OR (tw:(zitazonium)) OR (tw:(soltamox)) OR (tw:(ici-47699)) OR (tw:(ici-46474)) OR (tw:(ici46474)) OR (tw:(ici47699)) OR (tw:(ici-46,474)) OR (tw:(ici46,474)))) OR (af:((mh:(aromatase inhibitors)) OR (tw:(aromatase inhibitor)) OR (tw:(aromatase inhibitors)) OR (tw:(3,4-epoxy-5alpha-androstan-17-one)) OR (tw:(aminoglutethimide)) OR (tw:(anastrozole)) OR (tw:(exemestane)) OR (tw:(fadrozole)) OR (tw:(formestane)) OR (tw:(intraovarian peptides)) OR (tw:(letrozole)) OR (tw:(plomestane)) OR (tw:(vorozole)))) OR (af:((mh:(selective estrogen receptor modulators)) OR (tw:(selective estrogen receptor modulator)) OR (tw:(selective estrogen receptor modulators)) OR (tw:(serm)) OR (tw:(extend* "endocrine therapy")))) |
| #3 | ((tw:((mh:(neoplasm recurrence, local)) OR (mh:(disease-free survival)) OR ( mh:("Progression-Free Survival")) OR (mh:(recurrence)) OR ((("endocrine therapy" OR tamoxifen OR "aromatase inhibitor" OR "aromatase inhibitors")) AND resistan*) OR (tw:(disease-free survival)) OR (tw:(breast cancer free interval)) OR (tw:(recurren*)) ))) |
| #4 | ((tw:(pam50)) OR (tw:(pam AND "50")) OR (tw:(prosigna)) OR (tw:(endopredict)) OR (tw:(epclin)) OR (tw:(clinical treatment score)) OR (tw:(4-marker immunohistochemical score)) OR (tw:(ich4)) OR (tw:(mammaprint)) OR (tw:(oncomastr)) OR  (tw:(oncomas tr)) OR (tw:(omclin1)) OR (tw:(omclin2)) OR (tw:(oncotype dx)) OR (tw:(cts5)) OR (tw:(breast cancer index)) OR HOXB13$IL17BR OR ((tw:(treatment)) OR (tw:(recurrence)) OR (tw:(ror)) OR (tw:(decision)) OR (tw:(propensity)) OR (tw:(prognostic)) OR (tw:(predict*)) AND (tw:(score)) OR (tw:(panel)) OR (tw:(nomogram)) OR (tw:(nomograms))) OR ((tw:(biomarker* or marker*) AND (risk or survival or recurren* or failure or cancer-free or prevent* or prognos* or predict*)))) |
| #5 | #1 AND #2 AND #3 AND #4 |
| #6 | Limit #5 to English, 2021-present |

**Database:** **Embase <1974 to 2022 October 24>**  

| **#** | **Query** |
| --- | --- |
| 1 | exp breast tumor/ |
| 2 | breast neoplasm*.mp. |
| 3 | breast cancer.mp. |
| 4 | 1 or 2 or 3 |
| 5 | antineoplastic agents, hormonal/ |
| 6 | exp tamoxifen/ |
| 7 | tamoxifen.mp. |
| 8 | ICI-47699.mp. |
| 9 | Nolvadex.mp. |
| 10 | Novaldex.mp. |
| 11 | Tomaxithen.mp. |
| 12 | Zitazonium.mp. |
| 13 | ICI-46474.mp. |
| 14 | ICI46474.mp. |
| 15 | ICI47699.mp. |
| 16 | ICI-46,474.mp. |
| 17 | ICI46,474.mp. |
| 18 | Soltamox.mp. |
| 19 | 5 or 6 or 7 or 8 or 9 or 10 or 11 or 12 or 13 or 14 or 15 or 16 or 17 or 18 |
| 20 | exp aromatase inhibitor/ |
| 21 | Aromatase Inhibitor*.mp. |
| 22 | 3,4-epoxy-5alpha-androstan-17-one.mp. |
| 23 | Aminoglutethimide.mp. |
| 24 | Anastrozole.mp. |
| 25 | exemestane.mp. |
| 26 | Fadrozole.mp. |
| 27 | formestane.mp. |
| 28 | intraovarian peptides.mp. |
| 29 | Letrozole.mp. |
| 30 | plomestane.mp. |
| 31 | vorozole.mp. |
| 32 | 20 or 21 or 22 or 23 or 24 or 25 or 26 or 27 or 28 or 29 or 30 or 31 |
| 33 | exp selective estrogen receptor modulator/ |
| 34 | (exten* adj1 endocrine therapy).mp. |
| 35 | selective estrogen receptor modulator*.mp. |
| 36 | SERM.mp. |
| 37 | 33 or 34 or 35 or 36 |
| 38 | 19 or 32 or 37 |
| 39 | disease free survival/ |
| 40 | progression free survival/ |
| 41 | recurren*.mp. |
| 42 | recurrent disease/ |
| 43 | ((endocrine therapy or tamoxifen or aromatase inhibitor*) adj1 resistan*).mp. |
| 44 | disease free survival.mp. |
| 45 | breast cancer free interval.mp. |
| 46 | 39 or 40 or 41 or 42 or 43 or 44 or 45 |
| 47 | PAM50.mp. |
| 48 | (PAM adj1 "50").mp. |
| 49 | Prosigna.mp. |
| 50 | breast cancer index.mp. |
| 51 | endopredict.mp. |
| 52 | epclin.mp. |
| 53 | clinical treatment score.mp. |
| 54 | 4-marker immunohistochemical score.mp. |
| 55 | ICH4*.mp. |
| 56 | MammaPrint.mp. |
| 57 | Mammostrat.mp. |
| 58 | OncoMasTR.mp. |
| 59 | OncoMas TR.mp. |
| 60 | OMclin1.mp. |
| 61 | OMclin2.mp. |
| 62 | Oncotype DX.mp. |
| 63 | CTSS.mp. |
| 64 | HOXB13?IL17BR.mp. |
| 65 | (HOXB13 adj1 IL17BR).mp. |
| 66 | ((treatment or recurrence or ROR or decision or propensity or prognostic or predict*) adj3 (score or panel or nomogram)).mp. |
| 67 | ((biomarker* or marker*) adj2 (risk or survival or recurren* or failure or cancer-free or prevent* or prognos* or predict*)).mp. |
| 68 | 47 or 48 or 49 or 50 or 51 or 52 or 53 or 54 or 55 or 56 or 57 or 58 or 59 or 60 or 61 or 62 or 63 or 64 or 65 or 66 or 67 |
| 69 | 4 and 38 and 46 and 68 |
| 70 | limit 69 to (english language and yr="2006 -Current") |
| 71 | limit 70 to conference abstract status |
| 72 | 70 not 71 |
| 73 | limit 72 to dc=20211221-20221025 |
